# Fluid management in patients undergoing cardiac surgery: effects of an acetate- versus lactate-buffered balanced infusion solution on hemodynamic stability (HEMACETAT)

**DOI:** 10.1186/s13054-019-2423-8

**Published:** 2019-05-06

**Authors:** Carmen A. Pfortmueller, Livia Faeh, Martin Müller, Balthasar Eberle, Hansjörg Jenni, Björn Zante, Josef Prazak, Lars Englberger, Jukka Takala, Stephan M. Jakob

**Affiliations:** 1 Department of Intensive Care Medicine, Inselspital, Bern University Hospital, University of Bern, Freiburgstrasse 10, 3010 Bern, Switzerland; 2 Institute of Health Economics and Clinical Epidemiology, University Hospital of Cologne, Cologne, Germany; 3 Department of Emergency Medicine, Inselspital, Bern University Hospital, University of Bern, Bern, Switzerland; 4 Department of Anaesthesiology and Pain Medicine, Inselspital, Bern University Hospital, University of Bern, Bern, Switzerland; 5 Department of Cardiovascular Surgery, Inselspital, Bern University Hospital, University of Bern, Bern, Switzerland

**Keywords:** Fluid therapy, Crystalloid solutions, Cardiac surgical procedures, Hemodynamics, Randomized controlled trial, Vasoconstrictor agents, Perioperative period

## Abstract

**Background:**

Recent evidence suggests that acetate-buffered infusions result in better hemodynamic stabilization than 0.9% saline in patients undergoing major surgery. The choice of buffer in balanced crystalloid solutions may modify their hemodynamic effects. We therefore compared the inopressor requirements of Ringer’s acetate and lactate for perioperative fluid management in patients undergoing cardiac surgery.

**Methods:**

Using a randomized controlled double-blind design, we compared Ringer’s acetate (RA) to Ringer’s lactate (RL) with respect to the average rate of inopressor administered until postoperative hemodynamic stabilization was achieved. Secondary outcomes were the cumulative dose of inopressors, the duration of inopressor administration, the total fluid volume administered, and the changes in acid-base homeostasis. Patients undergoing elective valvular cardiac surgery were included. Patients with severe cardiac, renal, or liver disease were excluded from the study.

**Results:**

Seventy-five patients were randomly allocated to the RA arm, 73 to the RL. The hemodynamic profiles were comparable between the groups. The groups did not differ with respect to the average rate of inopressors (RA 2.1 mcg/kg/h, IQR 0.5–8.1 vs. RL 1.7 mcg/kg/h, IQR 0.7–8.2, *p* = 0.989). Cumulative doses of inopressors and time on individual and combined inopressors did not differ between the groups. No differences were found in acid-base parameters and their evolution over time.

**Conclusion:**

In this study, hemodynamic profiles of patients receiving Ringer’s lactate and Ringer’s acetate were comparable, and the evolution of acid-base parameters was similar. These study findings should be evaluated in larger, multi-center studies.

**Trial registration:**

Clinicaltrials.gov NCT02895659. Registered 16 September 2016.

**Electronic supplementary material:**

The online version of this article (10.1186/s13054-019-2423-8) contains supplementary material, which is available to authorized users.

## Introduction

Cardiovascular surgery generates a systemic inflammatory response that increases oxygen consumption and is associated with changes in cardiac output and oxygen delivery [1, 2]. Perioperative hemodynamic support is influenced by the patients’ underlying cardiac disease, the complexity of the surgical intervention, the inflammatory response to extracorporeal circulation, and the need for perioperative anticoagulation [3]. Patients often require several hours of postoperative hemodynamic support after cardiac surgery while still sedated and intubated on the intensive care unit (ICU) [4, 5], and frequently, they receive a substantial amount of intravenous fluid within a short time period.

Volume replacement strategies and type of fluid used in patients undergoing cardiac surgery have changed over the years [6]. Safety concerns regarding the use of synthetic colloid solutions in cardiac surgery patients [2, 7, 8] have led to increased use of crystalloid infusions [6, 9, 10].

Recently, two studies suggested that acetate-buffered crystalloid solutions result in better hemodynamic stabilization than 0.9% saline in patients undergoing major surgical procedures [11, 12]. However, the use of acetate-containing balance solutions has been criticized due to the risk of vasodilatation and metabolic alkalosis [13–18]. Patients undergoing cardiac surgery typically require several hours of hemodynamic support, and both acetate-buffered and lactate-buffered crystalloids are used in clinical practice.

We therefore hypothesized that the use of an acetate-based balanced crystalloid solution for perioperative fluid replacement in patients undergoing cardiac surgery would result in a lower average rate of inopressors, a lower cumulative dose of inopressors, a shorter total time on inopressors, and a lesser amount of fluid needed to achieve hemodynamic stability than the use of a lactate-buffered crystalloid solution.

## Methods

### Design

We conducted a randomized double-blind single-center clinical trial investigating the effect of Ringer’s acetate (RA) vs. Ringer’s lactate (RL) on hemodynamic stability and fluid requirements in patients undergoing cardiac surgery.

### Ethical considerations

The study was approved by the ethics committee of the Canton of Bern (2016-01039) and was registered in a clinical trial register (NCT02895659). Written preoperative informed consent was obtained from every patient included in the study.

### Setting

The study was conducted between December 1, 2016, and October 19, 2017, in the Department of Intensive Care Medicine of the Inselspital, Bern University Hospital, University of Bern, Bern, Switzerland.

### Study endpoints

The primary endpoint of this study was the average rate of inopressors (norepinephrine and epinephrine) per kilogram body weight-hour until hemodynamic stabilization. Secondary study endpoints were (i) cumulative inopressor dose, (ii) time on inopressors, (iii) cumulative dose of and time on inodilators and vasodilators, (iv) total amount of fluid administered until hemodynamic stabilization, and (v) occurrence of metabolic alkalosis. The study groups were compared with regard to the average rate of inopressor administration, the average rate of norepinephrine and epinephrine administration, the cumulative inopressor dose, the cumulative time on inopressors, the cumulative dose of and time on inodilators and vasodilators, the total amount of fluid administered until hemodynamic stabilization, and the occurrence of metabolic alkalosis. Further, fluid balance, urinary output, and blood loss were defined as outcomes post hoc. Additionally, patient characteristics and postoperative complications were evaluated. Metabolic alkalosis was defined as pH > 7.45 and bicarbonate > 26 mmol/L. Alkalemia was defined as pH > 7.45.

### Time to hemodynamic stabilization: definition of the study period

We aimed to investigate the perioperative period until achievement of hemodynamic stabilization. The study period commenced with the induction of general anesthesia and ended when hemodynamic stabilization was achieved. This point in clinical recovery was defined in the following way: (a) successful extubation and free from vasoactive support; (b) intubated with inopressor agents, but inopressor dose either weaned entirely or unchanged for > 8 h or (c) 72 h after ICU admission. Thereafter, follow-up continued for secondary endpoints for the duration of the hospital stay. The study fluid was not restarted once the initial stabilization was achieved.

The following is the rationale for this definition: the typical hemodynamic problems after cardiac surgery include reduced intravascular blood volume accompanied by peripheral vasoconstriction, low cardiac output (with or without hypotension) due to compromised cardiac function, and/or preload, and major changes in vascular tone [19, 20]. All these may be augmented due to heart-lung interactions during weaning and due to acute hemodynamic changes related to extubation. Our clinical guidelines recommend extubation as soon as the patient is awake and hemodynamically stable. Extubation is usually postponed if the patient needs frequent adjustment of vasoactive drugs and or volume substitution. We therefore took “successful extubation and free from vasoactive drugs” to imply hemodynamic stability and considered patients who remained intubated without hemodynamic support or did not need any change in the hemodynamic management for > 8 h as stable.

### Patients

**Fig. 1:**
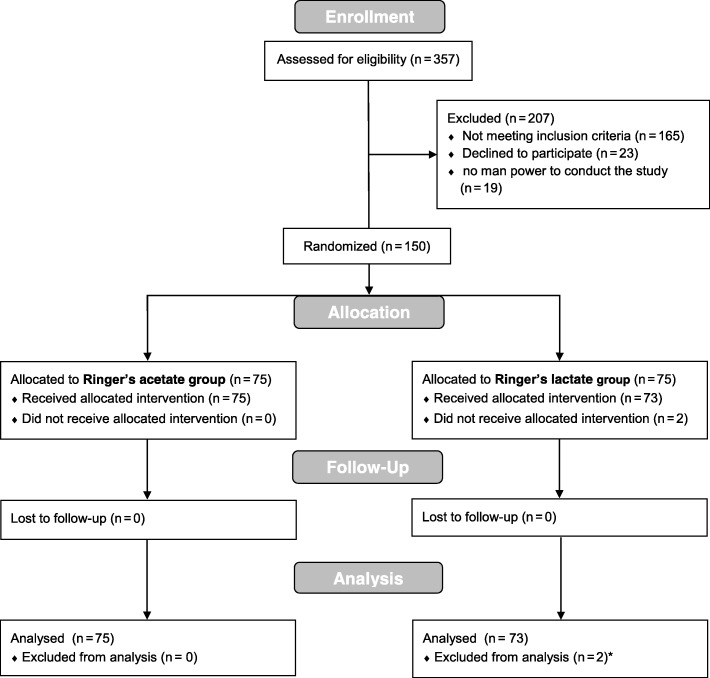
CONSORT flowchart

A CONSORT flow chart is provided in Fig. 1. Patients were eligible for the study if they were scheduled to undergo an elective open surgical single- or double-valve procedure, combined valve and coronary bypass surgery, or combined valve and proximal aortic surgery.

We included routine cardiac surgery patients with no severe comorbidities. Thus, the exclusion criteria were as follows: age less than 18 or older than 80 years, pre-existing severely impaired cardiac or renal function (EF < 30%; eGFR < 30 mL/min/1.73 m^2^), pre-existing anemia necessitating a cardiopulmonary bypass (CPB) circuit prime with red cell concentrate, chronic inflammatory diseases, on long-term steroid medication, chronic liver disease (bilirubin > 3 mg.dl^−1^), active infection or sepsis, emergency or redo surgery, planned use of minimal extracorporeal circuits (MECC) or of early (in the OR) extubation protocols, and patients with restrictions to full therapy. Isolated CABG surgery was excluded since minimized extracorporeal circulation and a fast-track regimen are used in our center for this type of cardiac surgery. This results in considerably less exposure to the intervention (crystalloid load) than the use of conventional CPB as in valve surgery.

### Randomization and material

Computerized randomization was performed in blocks of 20 patients. Concealment used the opaque sealed envelope method. After informed consent was obtained, patients were randomly allocated to receive either RA (Fresenius Kabi®, Switzerland GmbH; theoretical osmolarity 291 mosml/kg, containing sodium 137 mmol/L, potassium 4.0 mmol/L, chloride 110 mmol/L, calcium 1.65 mmol/L, magnesium 1.25 mmol/L, acetate 36.8 mmol/L) or RL (Fresenius Kabi®, Switzerland GmbH; theoretical osmolarity 278 mosm/L, sodium 130.9 mmol/L, potassium 5.4 mmol/L, chloride 111.7 mmol/L, calcium 1.84 mmol/L, lactate 28.3 mmol/L). For each patient, a box with concealed bags of study infusion solution was prepared by a study nurse not involved in the study or patient care and was handed over to both the anesthesiologist and the perfusionist prior to the start of anesthesia. Postoperatively, the box accompanied the patient to the ICU for further study fluid administration until hemodynamic stabilization.

### Study conduct

All patients underwent monitoring of oxygen saturation, heart rate, invasive arterial blood pressure, electrocardiography, respiratory gas analysis, temperature, urine output, central venous pressure, intraoperative transesophageal echocardiography, processed EEG, and postoperative peripheral temperature according to institutional routine. Maintenance of anesthesia, hemodynamic and perfusion management, surgical procedures, and cardiopulmonary bypass weaning were performed according to the departmental standard operating procedures (SOP). All patients were transferred sedated and ventilated from the OR to the ICU.

### Hemodynamic management

Patients received either RL or RA for intravenous fluid resuscitation according to their group allocation. The cardiopulmonary bypass circuit was also exclusively primed with the allocated study fluid. No colloid solutions were used. Hemodynamic and fluid management was guided by TEE and dynamic assessment of both fluid responsiveness and filling pressures, with staged escalation towards vasoactive and/or inotropic and/or mechanical support in case of persistent low cardiac output (mean arterial pressure (MAP) < 60 mmHg, CCI < 2.2 L/min/m^2^, S_mv_O_2_ < 65%, and/or lactate levels > 2.4 mmol/L). The choice of specific agents and interventions was left to the discretion of the attending physician specialists.

Following ICU admission, hemodynamic management [21] was guided using the following targets: MAP 60–90 mmHg, CVP target < 12 mmHg, oxygen saturation > 96%, and heart rate 60–110/min. Further, normalization of peripheral temperature, diuresis (target > 0.5 mL/kgBW/h), and arterial blood lactate concentration < 2.0 mmol/L were expected as the response to treatment. During mechanical ventilation, the pCO2 was kept at < 40 mmHg. Since patients after cardiac surgery are vasoconstricted, intravenous fluids were given to restore intravascular volume during vasodilation, to normalize peripheral perfusion, and to allow weaning from vasopressors. If patients were not responding as expected, echocardiography, pulmonary artery catheterization, or both were used to determine the underlying cause. The diagnosis and treatment of specific hemodynamic problems was at the discretion of the intensivist in charge of the patient.

### Statistical analysis

The statistical analysis was performed with Stata® 13.1 (StataCorp, College Station, TX, USA). All randomized patients who received the study fluid were included in the analysis according to a modified intention-to-treat approach [22]. Normal distribution of continuous variables was tested with the Shapiro-Wilk test. Normally distributed variables are presented with mean and standard deviation. Skewed and ordinal variables are presented as medians with interquartile ranges (IQR).

Normally distributed interval and ordinal variables were compared using the unpaired *t* test and skewed variables using the Wilcoxon rank sum test. Comparisons of categorical variables were performed using the chi-square test or Fisher’s exact test, as appropriate [23].

The procedure for analyzing the potential baseline differences between the groups in case of a significant group effect was log-linear regression analysis. All baseline characteristics with a *p* value of < 0.1 were included. In addition, further confounders related to increased inopressor use such as aortic insufficiency were also forced into the model. For the purpose of this analysis, skewed outcomes were log-transformed; thus, the exponentiated coefficients correspond to the geometric mean ratio of the outcome.

A mixed restricted cubic spline model was used for the evaluation of changes in hemodynamics and in acid-base homeostasis, i.e., mean arterial pressure, peripheral temperature, urinary output, base access, pH, potassium, and lactate over time between the study groups. Missing values were imputed with the use of the last observation carried forward method for measurements made after baseline.

*p* values less than 0.05 were considered statistically significant. Figures were drawn using Stata®.

## Results

**Fig. 2:**
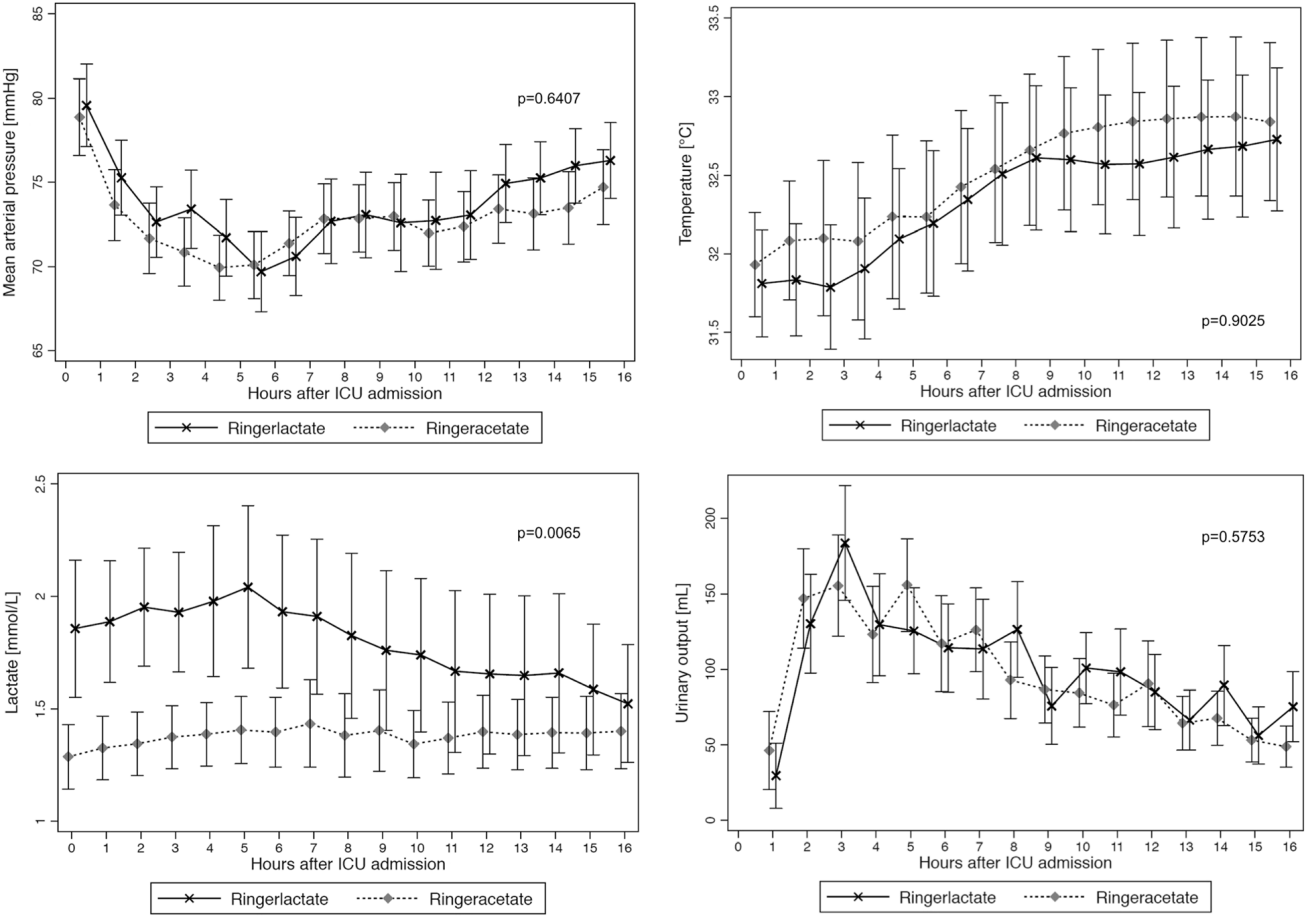
Hemodynamic profile for the Ringer’s acetate and the Ringer’s lactate group

Out of 357 screened patients, 150 gave consent and were randomized, and 148 patients actually received the study intervention (see Fig. 1). Seventy-five patients were randomized to the RA group and 73 patients to the RL group. Patients’ characteristics are given in Table 1. In the RL group, more patients underwent a composite graft operation (*n* = 16, 21.9% vs. *n* = 4, 5.3%), whereas in the RA group, more patients received a single-valve replacement (*n* = 59, 78.7% vs. *n* = 48, 65.8%). The number of patients undergoing hypothermic cardiac arrest was also higher in the RL group (*p* = 0.004). The evolution of blood pressure, peripheral temperature, and urinary output was similar in the Ringer’s acetate and the Ringer’s lactate groups (Fig. 2).

**Table 1:** Patient demographics

Characteristics	Total (*n* = 148)	Ringer’s acetate (*n* = 75)	Ringer’s lactate (*n* = 73)	*p* value*
Sex, *n* (%)				
Male	112 (75.7)	56 (74.7)	56 (76.7)	0.772
Female	36 (24.3)	19 (25.3)	17 (23.3)	
Age [years], med (IQR)	67.5 (58.0–72.5)	66.0 (58.0–72.0)	68.0 (59.0–73.0)	0.631
ASA PS [class], *n* (%)				1.000
3	3 (2.0)	2 (2.7)	1 (1.4)	
4	145 (98.0)	73 (97.3)	72 (98.6)	
Euroscore, med (IQR)	18 (17–19)	18 (17–19)	18 (16–19)	0.542
SAPS, med (IQR)	54 (44–62)	54 (46–62)	54 (42–62)	0.945
NYHA [grade], *n* (%)				0.167
0	11 (7.5)	5 (6.8)	6 (8.2)	
1	34 (23.1)	18 (24.3)	16 (21.9)	
2	69 (46.9)	34 (46.0)	35 (48.0)	
3	28 (19.1)	17 (23.0)	11 (15.1)	
4	5 (3.4)	0 (0.0)	5 (6.9)	
Preoperative ejection fraction [%], med (IQR)	60 (55–65)	60 (55–65)	60 (55–65)	0.795
Preoperative eGFR [mL/min], med (IQR)	70 (53–85)	73 (51–87)	67 (54–83)	0.507
Type of surgery, *n* (%)				
Composite graft (+valve^#^)	20 (13.5)	4 (5.3)	16 (21.9)	0.004
Other	128 (86.5)	71 (94.7)	57 (78.1)	
Single valve	107 (72.3)	59 (78.7)	48 (65.8)	
Single valve + CABG	12 (8.1)	5 (6.7)	7 (9.6	
Double valve	6 (4.1)	4 (5.3)	2 (2.7)	
Double valve + CABG	1 (0.7)	1 (1.3)	0 (0.0	
Triple valve	2 (1.4)	2 (2.7)	0 (0.0)	
Duration anesthesia [min], med (IQR)	310 (266–362)	305 (266–353)	310 (264–364)	0.937
Duration surgery [min], med (IQR)	202 (170–251)	205 (176–251)	192 (170–264)	0.812
Aortic cross clamp time [min], med (IQR)	70 (56–90)	67 (53–86)	73 (58–97)	0.172
Hypothermic circulatory arrest n (%)	17 (11.56)	3 (4.00)	14 (19.18)	0.004
Red blood cell transfusions [mL]°, med (IQR)	340 (220–550)	333 (223–575)	350 (174–550)	0.788
Time until initial hemodynamic stabilization [h], med (IQR)	12.06 (9.7–13.9)	12.08 (10.0–13.4)	12.05 (9.1–14.1)	0.649

*Wilcoxon rank sum test for continuous variables and chi-square test

°Fisher’s exact test for categorical variables between the Ringer’s acetate and the Ringer’s lactate study groups including autologous retransfusion and red cell concentrates

^#^One patient in the Ringer’s lactate group

Primary and secondary study endpoints with respect to the vasoactive medication are given in Additional file 1 and Table 2.

**Table 2:** Primary and secondary endpoints—vasoactive medication

Primary endpoint	Ringer’s acetate (*n* = 75), median (IQR)	Ringer’s lactate (*n* = 73), median (IQR)	*p* value
Average rate of inopressors (norepinephrine and epinephrine) [μg/kg/h] until hemodynamic stabilization	2.1 (0.5–8.1)	1.7 (0.7–8.2)	0. 989
Secondary endpoints—vasoactive medication			
Average rate of norepinephrine [μg/kg/h] until hemodynamic stabilization	1.8 (0.5–6.7)	1.5 (0.6–4.7)	0.672
Average rate of epinephrine [μg/kg/h]^§^ per hour on epinephrine	0.2 (0.1–9.3)	4.5 (0.4–33.0)	0.047
Average rate of inopressors (norepinephrine and epinephrine) [μg/kg/h] per hour on inopressors	4.1 (1.0–11.8)	3.7 (1.6–12.1)	0.959
Average rate of norepinephrine [μg/kg/h]^#^ per hour on norepinephrine	4.1 (1.1–11.7)	3.4 (1.5–9.5)	0.907
Average rate of epinephrine [μg/kg/h]^**§**^ per hour on epinephrine	0.2 (0.1–9.3)	4.5 (0.4–33.0)	0.047
Cumulative dose of inopressors [μg/kg]	22 (5–83)	20 (7–114)	0.928
Cumulative dose of norepinephrine [μg/kg]	19 (5–71)	16 (6–61)	0.726
Cumulative dose of epinephrine [μg/kg]	22 (5–83)	20 (7–114)	0.928
Time on inopressors [h]°	4.8 (4.1–10.7)	6.1 (4.0–10.6)	0.505
Time on norepinephrine [h]^#^°	4.8 (4.1–6.4)	4.5 (3.7–7.4)	0.836
Time on epinephrine [h]*°	0.0 (0.0–0.0)	0.0 (0.0–3.4)	0.209
Cumulative dose of inodilators, ICU [μg]	0 (0.0–0.0)	0 (0.0–0.0)	0.375
Time on inodilators, ICU [min]	0 (0.0–0.0)	0 (0.0–0.0)	0.386
Cumulative dose of vasodilators [mg]	0 (0.0–2.8)	0 (0.0–4.2)	0.844
Time on vasodilators, ICU [h]	0 (0.0–1.7)	0 (0.0–6.4)	0.726
Secondary endpoints—fluids			
Total amount of study fluid received (mL)	6677 (5325–8479)	6104 (4769–7855)	0.272
Total amount of fluid received other than study fluid during study period (mL)	0 (0–0)	0 (0–0)	0.976
Total amount of fluid received after initial hemodynamic stabilization until ICU discharge (mL)	289 (104–972)	255 (121–631)	0.711
Total amount of fluid received from the start of anesthesia to ICU discharge (mL)	7189 (5622–9120)	6644 (5400–8379)	0.234

*Not including four patients who received one single-bolus injection of epinephrine only

^#^Not including one patient who received one single-bolus injection of norepinephrine only

°From intubation to end of study period defined

**Fig. 3:**
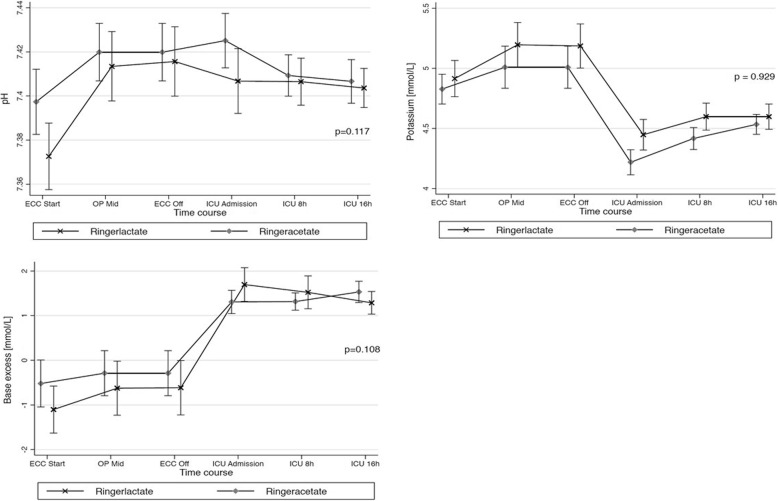
Acid-base homeostasis—profile of Ringer’s acetate and Ringer’s lactate

Patients in the RA group did not differ from the RL group with respect to the average rate of inopressors (2.1 mcg/kg/h, IQR 0.5–8.1 and 1.7 mcg/kg/h, IQR 0.7–8.2, respectively, *p* = 0.989). In the log-linear regression model adjusted for the baseline differences between the groups, no significant group effect was found with respect to the primary outcome (*p* = 0.494). Data on fluids is presented in Table 2. Fluid balance, urinary output, and blood loss did not differ significantly between the groups (data not shown). Changes in acid-base status over time are shown in Fig. 3. No difference in the occurrence of metabolic acidosis (*p* = 0.327) or alkalemia (*p* = 0.681) was noted between the groups. Postoperative outcomes are given in Table 3. Except for the occurrence of postoperative arrhythmia (*p* = 0.008), no significant differences in postoperative outcomes were noted (*p* all > 0.05).

**Table 3:** Postoperative outcomes

Characteristics	Ringer’s acetate (*n* = 75)	Ringer’s lactate (*n* = 73)	*p* value*
Occurrence of arrhythmia, *n* (%)	24 (32.0)	39 (53.4)	0.008
Acute kidney injury, *n* (%)^#^	11 (14.7)	10 (13.7)	0.866
Risk	10	8	0.901
Injury	1	1	
Failure	0	1	
New neurological deficit, med (IQR)	4 (5.3)	7 (9.6)	0.324
Critical illness polyneuropathy	0	1	
Ischemic stroke	4	5	
Peripheral nerve lesion	0	1	
Coronary angiography, *n* (%)	2 (2.7)	5 (6.9)	0.272
Postoperative drainage output	950 (550–1200)	1000 (560–1320)	0.415
Received blood product transfusion, *n* (%)	24 (32.0)	19 (26.0)	0.424
Postoperative infection, *n* (%)	4 (5.3)	3 (4.1)	1.000
Other complications, *n* (%)	8 (10.7)	4 (5.5)	0.368
Delirium	1	0	
Pneumothorax	2	0	
Significant pleural effusion	2	1	
Skin rash	1	1	
Transient hypoxemia	2	2	
Surgical re-exploration needed, *n* (%)	2 (2.7)	6 (8.2)	0.166
Need for postoperative mechanical circulatory support, *n* (%)	0(0.0)	2 (2.7)^+^	0.242
Length of stay [days], med (IQR)			
ICU	0.9 (0.7–0.9)	0.9 (0.8–0.9)	0.494
IMC	0.0 (0.0–0.0)	0.0 (0.0–1.0)	0.362
Hospital	9.0 (8.0–12.0)	9.0 (8.0–10.2)	0.425
In-hospital mortality	0	0	

^#^According to the RIFLE criteria

*Wilcoxon rank sum test for continuous and Fisher’s exact test for categorical variables between the Ringer’s acetate and Ringer’s lactate study groups

^+^One patient arrived with IABP from the OR, and one patient needed veno-arterial ECMO support in ICU

### Sensitivity analysis: inopressors—norepinephrine and epinephrine

All patients received norepinephrine infusions (*n* = 148, 100%), and 41 patients (27.7%) received epinephrine infusions at some time during the perioperative period (both *p* > 0.05 between the groups). The average norepinephrine rate did not differ between the RA group (median 4.1 mcg/kg/h, IQR 1.1–11.7) and the RL group (median 3.4 mcg/kg/h, IQR 1.5–9.5, *p* = 0.907). However, patients in the RL group received significantly more epinephrine on average compared to the RA group (4.5 μg/kg/h, IQR 0.4–33.0 vs. 0.2 μg/kg/h, IQR 0.1–9.3, *p* = 0.047). The significant group effect with regard to the average rate of epinephrine was persistent when using log-linear regression analysis controlled for composite graft operations, deep hypothermic circulatory arrest, and valvular regurgitation (geometric mean ratio 9.9, 95% CI 1.3, 78.3, *p* = 0.030).

If only the period in the ICU is considered, more patients in the Ringer’s lactate group received epinephrine infusions (14 [19.2%] vs. 6 [8.0%], *p* = 0.047).

## Discussion

The main finding of this study is that acetate-buffered balanced Ringer’s solution does not differ significantly from lactate-buffered Ringer’s solution with respect to postoperative hemodynamic stability and phamacological support with inotropic agents in patients scheduled for elective cardiac valve surgery. Also, we observed no difference in the cumulative amount of perioperative i.v. fluids necessary or in acid-base profiles. A small but significant increased average rate of epinephrine was noted in the group with lactate-buffered infusion. The difference in rate was very small, however, and likely of no clinical importance.

Previous observations in humans suggested that the choice of crystalloid fluid might influence the requirements for perioperative vasoactive agents [11, 12, 24]. In these studies, the use of 0.9% saline was significantly associated with more frequent vasoactive medication when compared to an acetate-buffered infusion group. This led to the hypothesis that either the high chloride load of 0.9% saline or the potentially beneficial effects of acetate on the cardiovascular system, seen in the animal model, may be responsible for the difference seen in these studies [12]. Therefore, we designed the current study comparing two chloride-reduced infusion solutions, one of which is acetate-buffered.

Based on our results, we now reject our hypothesis that acetate-buffered Ringer’s solution is superior to Ringer’s lactate solution with respect to perioperative hemodynamic stability.

The hemodynamic effects of acetate are controversial. Some studies have reported a decline in blood pressure after sodium acetate infusion [25–28], whereas others observed stable [29, 30] or even increasing blood pressure [16]. However, most of this evidence originates from the investigations of either sodium acetate infusion or acetate-buffered dialysis, where the acetate load is much higher than in fluid replacement with acetate-buffered Ringer’s solution. Therefore, such findings are not generalizable to populations receiving perioperative fluid therapy with acetate-buffered infusions [31]. In fact, the few existing animal and human studies investigating acetate-buffered crystalloid infusions noted potentially beneficial effects on cardiac function [14, 16, 32].

In our study, the hemodynamic profiles of patient groups receiving either RA or RL did not differ significantly, even though large quantities of i.v. fluid were administered. RA therefore appears to be a feasible alternative to RL for fluid resuscitation in the critically ill in terms of hemodynamic effects.

The lack of difference between the two study arms with respect to hemodynamic stabilization in comparison with earlier trials might also be attributed to the reduced chloride load of buffered infusions when compared to saline. Chloride excess was linked to adverse hemodynamic outcomes in several previous human and experimental studies [33–36]. However, the data suggesting that the use of 0.9% saline leads to hemodynamic effects are limited and certainly need to be verified in a larger cohort before any definitive conclusions can be drawn.

Acetate and lactate are both weak acids that are converted into bicarbonate [31]. Lactate can be utilized by multiple pathways and is unlikely to produce acute changes in acid-base balance [37]. In contrast, acetate-buffered solutions may produce metabolic alkalosis due to rapid production of bicarbonate from acetate [13, 15, 38, 39].

However, a recent systematic review of studies comparing acetate-buffered solution to other crystalloids found that major increases in bicarbonate with acetate-buffered solutions were rarely observed [31]. Our study shows that even in patients undergoing cardiac surgery who receive large quantities of intravenous fluid within a short time period, the use of an acetate-buffered infusion solution did not result in a difference in metabolic alkalosis or alkalemia between the study groups.

Postoperative arrhythmias were recorded significantly more often in patients in the RL group than in patients in the RA group. Electrolyte blood concentrations were similar. Potential explanations are the differences in case mix, more frequent use of epinephrine in the RL group, and/or pre-existing but not documented differences in atrial geometry; however, this needs to be investigated further.

In our study cohort, 7.4% of the patients suffered from new postoperative neurological deficit. Cardiac valve procedures per se are a known risk factor for postoperative neurologic deficits in patients undergoing cardiac surgery [40]. In addition, the high proportion of patients that received an aortic or composite-graft procedure, the advanced age, and the high incidence of perioperative arrhythmia, all of which are well-known risk factors for perioperative strokes, might explain the proportion of 6.1% in our study population suffered from postoperative new stroke [40, 41].

The results of this study show that RA appears to be a feasible alternative to RL for perioperative fluid resuscitation in the critically ill, even when large quantities are needed. This could have implications for clinical practice.

The limitations of our study are its single-center design and the exclusion of patients with more severe cardiac and renal impairment. The former were excluded due to the higher need for perioperative inopressors in this population as a result of the underlying cardiac disease; that latter were excluded because patients with severe kidney dysfunction have an impaired ability to react to changes in acid-base homeostasis. Another limitation might arise from the fact that patients only received the study fluid until hemodynamic stabilization and not until ICU discharge. However, in our ICU, we give maintenance fluid after hemodynamic stabilization/extubation mainly for potassium substitution to prevent arrhythmia, and the amount is generally negligible. Further limitations arise from the imbalanced distribution of ascending aortic procedures between the groups, from the exclusion of patients undergoing only isolated CABG surgery, and due to the limited sample size. In addition, our study was designed as a pragmatic trial comparing the two solutions in the context of both groups receiving usual care. We cannot address the possible specific hemodynamic effects (e.g., vasodilatation) of either solution.

Further confirmatory studies are certainly warranted in the future.

## Conclusion

In this study, in patients undergoing cardiac valve surgery, hemodynamic profiles and inopressor requirements were similar in patients receiving Ringer’s lactate and Ringer’s acetate for perioperative fluid resuscitation. Between-group differences in metabolic alkalosis commonly associated with larger loads of acetate infusion were not observed in this study. In this trial, Ringer’s acetate solution appears to be an acceptable alternative to Ringer’s lactate solution for perioperative fluid resuscitation in cardiac surgery. Our results need to be verified in a larger cohort, however.

## Additional file

Additional file 1: **Table S1.** Primary and secondary endpoints—anesthesia versus ICU. (DOCX 15 kb)

## Abbreviations

ICU: Intensive care unit
MAP: Mean arterial pressure
OR: Operating room
RA: Ringer’s acetate
RL: Ringer’s lactate
SOP: Standard operating procedures
