# Supplementary material for: Fluid management in patients undergoing cardiac surgery: effects of an acetate- versus lactate-buffered balanced infusion solution on hemodynamic stability (HEMACETAT)
Source: Crit Care. 2019 May 6;23:159. doi: 10.1186/s13054-019-2423-8 (PMC6503387; doi:10.1186/s13054-019-2423-8)
Supplement: Supplementary file 1 — Table S1. Primary and secondary endpoints—anesthesia versus ICU. (DOCX 15 kb) [file 13054_2019_2423_MOESM1_ESM.docx]

**Table S1.** Primary and secondary endpoints – anesthesia versus ICU

| **anesthesia** | | **Acetated Ringer’s**  **(n= 75)** | **Lactated Ringer’s**  **(n=73)** | **p value** |
| --- | --- | --- | --- | --- |
|  |  | median (IQR) | median (IQR) |  |
|  | **Cumulative dose of inopressors [µg/Kg]** | 6.8 (3.6-10.9) | 6.8 (2.9-10.2) | 0.669 |
|  | Cumulative norepinephrine dose [µg/Kg] | 6.6 (3.6-9.8) | 6.2 (2.9-9.1) | 0.381 |
|  | Cumulative epinephrine dose [µg/Kg] | 0.0 (0.0-0.0) | 0.0 (0.0-0.4) | 0.264 |
|  |  |  |  |  |
|  | **Total amount of study fluid received (ml)** | 4200 (3200-5000) | 4,100 (3200-4300) | 0.355 |
| **ICU** | | | | |
|  | **Cumulative dose of inopressors [µg/Kg]** | 13.1 (0-77.9) | 13.7 (0-106.9) | 0.790 |
|  | Cumulative norepinephrine dose [µg/Kg] | 13.1 (0-77.9) | 13.7 (0-106.9) | 0.790 |
|  | Cumulative epinephrine dose [µg/Kg] | 0.0 (0.0-0.0) | 0.0 (0.0-0.0) | 0.261 |
|  |  |  |  |  |
|  | **Total amount of study fluid received (ml)** | 2372 (1301-3964) | 2,292 (988-3971) | 0.788 |
